# Morphological evidence for dopamine interactions with pallidal neurons in primates

**DOI:** 10.3389/fnana.2015.00111

**Published:** 2015-08-11

**Authors:** Lara Eid, Martin Parent

**Affiliations:** Department of Psychiatry and Neuroscience, Centre de Recherche de l'Institut Universitaire en Santé Mentale de Québec, Université LavalQuebec City, QC, Canada

**Keywords:** basal ganglia, globus pallidus, squirrel monkey, electron microscopy, stereology, pallidum, substantia nigra, TH immunohistochemistry

## Abstract

The external (GPe) and internal (GPi) segments of the primate globus pallidus receive dopamine (DA) axonal projections arising mainly from the substantia nigra pars compacta and this innervation is here described based on tyrosine hydroxylase (TH) immunohistochemical observations gathered in the squirrel monkey (*Saimiri sciureus*). At the light microscopic level, unbiased stereological quantification of TH positive (+) axon varicosities reveals a similar density of innervation in the GPe (0.19 ± 0.02 × 10^6^ axon varicosities/mm^3^ of tissue) and GPi (0.17 ± 0.01 × 10^6^), but regional variations occur in the anteroposterior and dorsoventral axes in both GPe and GPi and along the mediolateral plane in the GPe. Estimation of the neuronal population in the GPe (3.47 ± 0.15 × 10^3^ neurons/mm^3^) and GPi (2.69 ± 0.18 × 10^3^) yields a mean ratio of, respectively, 28 ± 3 and 68 ± 15 TH+ axon varicosities/pallidal neuron. At the electron microscopic level, TH+ axon varicosities in the GPe appear significantly smaller than those in the GPi and very few TH+ axon varicosities are engaged in synaptic contacts in the GPe (17 ± 3%) and the GPi (15 ± 4%) compared to their unlabeled counterparts (77 ± 6 and 50 ± 12%, respectively). Genuine synaptic contacts made by TH+ axon varicosities in the GPe and GPi are of the symmetrical and asymmetrical type. Such synaptic contacts together with the presence of numerous synaptic vesicles in all TH+ axon varicosities observed in the GPe and GPi support the functionality of the DA pallidal innervation. By virtue of its predominantly volumic mode of action, DA appears to exert a key modulatory effect upon pallidal neurons in concert with the more direct GABAergic inhibitory and glutamatergic excitatory actions of the striatum and subthalamic nucleus. We argue that the DA pallidal innervation plays a major role in the functional organization of the primate basal ganglia under both normal and pathological conditions.

## 1. Introduction

The functional importance of the dopamine (DA) neurons located in the brainstem substantia nigra pars compacta (SNc) is underlined by their role in the pathophysiology of Parkinson's disease (Penney and Young, 1983; Albin et al., 1989; Goto et al., 1989; Smith and Kieval, 2000; Rommelfanger and Wichmann, 2010; Benazzouz et al., 2014). Axons of these neurons arborize extensively in the striatum (Fallon and Moore, 1978; Lindvall and Björklund, 1979; Cossette et al., 1999; Prensa et al., 2000) where they modulate the activity of the medium spiny neurons that project to the external (GPe) and internal (GPi) segments of the globus pallidus, as well as to the substantia nigra pars reticulata (Penney and Young, 1983; Albin et al., 1989; Parent and Hazrati, 1995). Thus, the nigral neurons play a substantial role in motor learning and related behaviors (Matsumoto et al., 1999; Kempf et al., 2007; Tremblay et al., 2010).

In addition to the well-established nigrostriatal pathway, evidence of a significant DA input to the GPe and GPi was gathered in both primates (Parent and Smith, 1987; Lavoie et al., 1989; Smith et al., 1989; Charara and Parent, 1994; Cossette et al., 1999; Hedreen, 1999; Jan et al., 2000; Prensa et al., 2000; Smith and Villalba, 2008) and rodents (Fallon and Moore, 1978; Lindvall and Björklund, 1979; Rodrigo et al., 1998). The primate pallidum is biochemically enriched in DA (Pifl et al., 1990), the neurotransmitter levels being nearly six times higher in the GPe than in the GPi (Rajput et al., 2008). The presence of DA receptors belonging to either the D_1_-like family (comprising the D_1_ and D_5_ receptor types) or the D_2_-like family (including D_2_, D_3_, and D_4_ receptor types) has been documented at pallidal levels, with D_1_ and D_2_ receptor types predominating in the GPi and GPe, respectively (Richfield et al., 1987). In both pallidal segments, the majority of DA receptors are located presynaptically on the striatopallidal axons (Kliem et al., 2007; Hadipour-Niktarash et al., 2012). Among these axons, those terminating in the GPe, which is reciprocally linked with the subthalamic nucleus, are believed to derive from medium spiny striatal neurons expressing the D_2_ receptor type, whereas those that arborize in the GPi, a major output structure of the basal ganglia, are thought to emerge from medium spiny striatal neurons expressing the D_1_ receptor type (Yung et al., 1995; Gerfen and Bolam, 2010). There are also data supporting the presence of D_2_ or D_1_ receptors expressed postsynaptically by GPe or GPi neurons (see Smith and Villalba, 2008; Rommelfanger and Wichmann, 2010). Moreover, ultrastructural investigations have reported the existence of synaptic contacts between DA axon terminals and pallidal dendrites in the rat (Rodrigo et al., 1998) and monkey (Smith and Kieval, 2000), providing further support of a direct DA modulation of pallidal activity. On a more functional point of vue, injections of D_1_ or D_2_ receptor agonists or antagonists were shown to either increase or decrease the firing rates of pallidal neurons in rats (Qi and Chen, 2011) and monkeys (Kliem et al., 2007; Hadipour-Niktarash et al., 2012).

The DA innervation of the pallidum has been examined at the light microscopic level in monkeys (Parent and Smith, 1987; Lavoie et al., 1989; Smith and Kieval, 2000) and humans (Jan et al., 2000). However, significant differences between human and non-human primates have been noted in regard to the topographical distribution and density of pallidal DA innervation. Such discrepancies are more likely the reflect of methodological variations rather than genuine interspecific differences, a demontrastion that would have necessitated the careful and uniform application of stringent stereological procedures. Moreover, although the existence of DA synaptic contacts in the primate pallidum has been alluded some time ago (Smith and Kieval, 2000), there has been yet no detailed morphological investigation of the DA innervation of the monkey GPe and GPi. Hence, in an attempt to complement our knowledge of how DA interacts with various components of the primate basal ganglia, we initiated a detailed light and electron microscopic immunohistochemical study of the DA innervation of the pallidum in the squirrel monkey. Efforts were made to compare the topographical distribution, the ultrastructural characteristics and the relational features of the DA axon terminals with intrinsic pallidal elements in both pallidal segments. We hope that such a comparison between pallidal segments will provide a better understanding of the role of DA within the GPe and GPi, two nuclei that are morphologically analogous but functionally highly dissimilar.

## 2. Materials and methods

### 2.1. Animals

Four adult male squirrel monkeys (*Saimiri sciureus*, Buckshire Corporation, Perkasie, PA, USA) weighing 952 ± 68 g were used to carry out this light and electron microscopy study. Animals were housed under a 12 h light-dark cycle, with food and water *ad libitum*. Our experimental protocol was approved by the “Comité de Protection des Animaux de l'Université Laval,” and all procedures involving animals and their care were made in accordance with the Canadian Council on Animal Care's Guide to the Care and Use of Experimental Animals (Ed2). Maximum efforts were made to minimize the number of animals used.

### 2.2. Tissue preparation

Animals were first deeply anesthesized with a mixture of ketamine (20 mg/kg, i.m.) and xylazine (4 mg/kg, i.m.), along with acepromazine (0.5 mg/kg, i.m.), and were then transcardially perfused following the exact same method as described in Eid et al. (2014). Brains were rapidly dissected out and postfixed by immersion in 4% PFA for 1 h at 4°C. The right hemispheres were cut along the coronal plane with a vibratome (Leica) into 50 μm-thick sections collected in sodium phosphate-buffered saline (PBS; 100 mM, pH 7.4). The left hemispheres were cut in different planes and sections were stored in an antifreezing solution made of 40% phosphate-buffered saline (PB, 50 mM), 30% glycerol (product no. G33-4, Fisher Scientific Company, Ottawa, ON, Canada) and 30% ethylene glycol (product no. E178-4, Fisher Scientific Company), and kept at −30°C for further experiments.

### 2.3. Antibodies

The monoclonal antibody used in the present immunohistochemical study was raised in mouse against tyrosine hydroxylase (TH; product no. 22941, ImmunoStar, Hudson, WI, USA), the catalytic enzyme for the conversion of tyrosine into the DA precursor dihydroxyphenylalanine (L-Dopa), isolated and purified from rat PC12 cells. Western blot in mouse brain tissue with this antibody showed a 60 kDa immunoreactive band typical of TH protein (Darmopil et al., 2008). Rodent and primate brain sections immunostained with this particular antibody displayed density and topography of axonal arborizations expected from catecholamine (DA and noradrenaline) neurons only, as it precisely matched that obtained from another anti-TH serum (Arluison et al., 1984) as well as from the same anti-TH serum (Jan et al., 2000; Fuchs and Hauber, 2004; Bernácer et al., 2012). Omitting primary or secondary antibody completely abolished immunostaining.

Because TH is also involved in the synthesis of noradrenaline, we performed a double immunofluorescence to confirm the findings of Gaspar et al. (1985) indicating that TH fibers observed in the globus pallidus are indeed DA and not noradrenergic. Hence, in addition to the TH antibody mentioned above, we used the rat monoclonal antibody against the dopamine transporter (DAT; product no. MAB369, EMD Millipore Corporation, Billerica, MA, USA). This particular DAT antibody was raised by isolating and purifying the N-terminus of human DAT fused to Glutathione S-transferase. It was characterized by Western blot and no cross-reactivity with the serotonin and norepinephrine transporters was observed in human brain tissue (Miller et al., 1997).

### 2.4. Assessment of the DA nature of the TH innervation

Two coronal sections from anterior (AP = 11.5 mm) and posterior (AP = 8.5 mm, according to interaural stereotaxic coordinates of Emmers and Akert, 1962) levels of the pallidum of one monkey were chosen to be processed for TH and DAT double immunofluorescence. All incubation steps were performed at room temperature, unless stated otherwise. To eliminate aldehyde bonds created by aldehyde fixation, free-floating sections were incubated in a solution of 0.5% NaBH_4_ diluted in PBS (30 min) followed by several rinses in PBS. They were then blocked for 1 h in a solution of PBS containing 2% normal horse serum, 2% normal goat serum and 0.3% Triton X-100. Sections were then incubated overnight (ON) in the same blocking solution to which dilutions of 1:1000 mouse anti-TH and 1:500 rat anti-DAT were added. After being thoroughly rinsed in PBS, sections were incubated for 2 h with biotinylated horse anti-mouse antibody (product no. BA 2000; Vector Laboratories, Burlingame, CA, USA) diluted 1:1000 in blocking solution, followed by more rinses in PBS. They were then incubated for another 2 h in the same blocking solution containing 1:200 dilutions of (i) Alexa Fluor 594 goat anti-rat (product no. A-11007; Molecular Probes, Life technologies, Burlington, ON, Canada) and (ii) DyLight 405 streptavidin (product no. 016-470-084; Jackson ImmunoResearch Laboratories Corporation, West Grove, PA, USA). Sections were rinsed in PBS, mounted on gelatin-coated slides, air-dried, and processed with autofluorescence eliminator reagent (product no. 2160; EMD Millipore Corporation), according to instructions provided by the manufacturer, after which they were coverslipped with Vectashield fluorescent mounting medium (product no. H-1400; Vector Laboratories).

Sections processed for TH and DAT double immunofluorescence were examined and imaged with a LSM 700 confocal microscope (Zeiss Canada) equipped with four solid-state lasers and a 63X/1.4 oil objective. The thorough examination of the doubly labeled sections containing the GPe and GPi revealed that virtually all TH-immunostained profiles within the confines of the pallidum were also DAT-immunoreactive, confirming their DA nature (see Supplementary Figure).

### 2.5. Stereology

#### 2.5.1. Immunohistochemistry

Free-floating brain sections from each of the four monkeys were first incubated as above in a 0.5% NaBH_4_ solution diluted in PBS for 30 min. Following several rinses in PBS, they were blocked for 1 h in a solution of PBS containing 2% normal horse serum and 0.5% Triton X-100. Sections were then incubated ON in the same blocking solution to which a 1:2000 dilution of mouse anti-TH was added, rinsed thoroughly in PBS and incubated for 2 h in the same blocking solution containing a 1:1000 dilution of the same biotinylated horse anti-mouse antibody described above. After rinses in PBS, sections were incubated for 1 h in avidin–biotine-peroxidase complex (ABC, product no. PK-4000; Vector Laboratories) diluted 1:100 in PBS. They were rinsed twice in PBS and once in Tris-buffered saline (TBS; 50 mM, pH 7.4) after which the bound peroxidase was revealed by incubating the sections for 4 min in a solution of TBS containing 0.05% 3,3′diaminobenzidine (product no. D5637; Sigma, St-Louis, MO, USA) and 0.005% H_2_O_2_. The reaction was stopped by several rinses in TBS followed by phosphate-buffered saline (PB; 100 mM, pH 7.4) and sections were mounted on gelatin-coated slides, air-dried, dehydrated in series of graded alcohol, cleared in toluene and coverslipped with Permount.

#### 2.5.2. TH-immunoreactive axon terminals

The stereological procedures used in this study are described in details elsewhere (Eid et al., 2013). In brief, TH-immunoreactive axon terminals in the GPe and GPi were examined with a light microscope and quantified using the unbiased stereological approach driven by the StereoInvestigator software (v.10.54, MicroBrightField, Colchester, CT, USA). For each monkey, eight equally-spaced coronal sections were selected across the entire GPe and GPi at an interval of 600 and 300 μm, respectively. The precise regional distribution of TH-immunoreactive axon terminals throughout the GPe and GPi was achieved by dividing each segment into eight sectors, according to the method described previously in Eid et al. (2013). Hence, each pallidal section was divided into dorsal, ventral, medial, and lateral sectors. The anteroposterior axis was divided in two by considering the first four coronal sections as representative of anterior sectors and the last four of posterior sectors, thus completing the eight sectors. The sampling of TH-immunoreactive axon varicosities was initiated by randomly placing a grid formed by 300 × 300 μm squares over the sections. At each intersection of the grid that fell into the sector, a 30 × 30 μm counting frame was drawn and examined with a 100X/1.30 oil-immersion objective. TH-immunoreactive axon varicosities, which appear as round or ovoid axonal dilation (>0.25 μm in transverse diameter) under the light microscope, were counted whenever one was encountered inside the counting frame, did not touch the exclusion lines and came into focus inside a 10 μm-thick optical disector centered in the section. The thickness of the mounted tissue was measured for each counting frame, yielding mean values of 20.1 ± 0.1 μm in the GPe and 20.0 ± 0.1 μm in the GPi. For each sector of the GPe and GPi, an average number of 201 ± 25 axon varicosities were counted and coefficients of error (Gunderson, *m* = 1 and 2^nd^ Schmitz–Hof) ranged between 0.04 and 0.18, except for one sector yielding 0.30. The density of TH innervation was obtained for each sector and for the entire GPe and GPi by using the total number of axon varicosities calculated by the optical disector and the volume estimated by Cavalieri's method, yielding values expressed in millions (10^6^) of axon varicosities per mm^3^ of tissue.

#### 2.5.3. Neuronal population in the monkey pallidum

The assessment of the GPe and GPi neuronal population was achieved according to the method used by Eid et al. (2013). In brief, adjacent coronal sections to those used for TH quantification were Nissl-stained in order to estimate the total neuronal population of each pallidal segment. Sections were mounted on gelatin-coated slides, air-dried, dehydrated in 70% ethanol (10 min), rehydrated in distilled water (5 min), and stained with cresyl violet (20 min). They were then dehydrated through a series of graded alcohols, cleared in toluene, and coverslipped with Permount.

The unbiased quantification was achieved by using the same stereological approach as described above, except that the grids were formed by 360 × 360 μm squares, the counting frame measured 200 × 200 μm and Nissl-stained neurons were examined with a 20X/0.70 objective through a 12 μm-thick optical disector centered in the section. Neurons were counted whenever the nucleolus came into focus inside the counting frame and did not touch the exclusion lines. Gunderson (*m* = 1) and 2^nd^ Schmitz–Hof coefficients of error yielded values ranging between 0.05 and 0.16 and the estimated neuronal population was used to calculate the number of TH-immunoreactive axon varicosities per pallidal neuron.

### 2.6. Electron microscopy

#### 2.6.1. Immunohistochemistry

Two sections from each monkey were chosen at the mid anteroposterior level of the pallidal complex (AP = 11.0 mm; Emmers and Akert, 1962) and were incubated as described above for light microscopy, i.e., with the same primary and secondary antibodies, with the exception that Triton X-100 was replaced by 0.5% cold fish gelatin. The secondary antibody was incubated for 1.5 h and ABC elite (product no. PK6100, Vector Laboratories) was used instead of standard ABC, with a 1.5 h incubation time. Sections were then incubated for 30 min in a 1% solution of OsO_4_ diluted in PB, followed by several rinses in PB. They were then dehydrated in graded ethanol series and in propylene oxide and flat-embedded in Durcupan (product no. 44611-14; Fluka, Buchs, Switzerland) to be processed and examined with the electron microscope.

#### 2.6.2. Preparation of electron microscopy samples

Quadrangular pieces were cut in the GPe and GPi of each monkey from flat-embedded TH-immunostained sections, glued on the tip of a resin block and cut ultrathin (~80 nm) with an ultramicrotome (Leica EM UC7). The ultrathin sections were collected on formvar-coated nickel slot grids or bare 150-mesh copper grids and stained with lead citrate. Grids were examined with a *Tecnai 12* transmission electron microscope (100 kV; Philips Electronic) equipped with an integrated Mega-View II digital camera (SIS, Germany). Axon varicosities were identified by their diameter > 0.25 μm and their content in synaptic vesicles, often associated with one or more mitochondria. Myelinated axons were readily identified by their high content in microtubules and by typical electron-dense myelin sheath observed around the axon. TH-immunoreactive axon varicosities and myelinated axons were randomly sampled at a working magnification of 11,500X by taking a picture every time such profile was encountered until 45 or more pictures were available for analysis in each pallidal segment, for each monkey.

#### 2.6.3. Fine morphological analysis of pallidal TH innervation

The fine morphological features of TH-immunoreactive axon varicosities and myelinated axons were analyzed with the public domain *ImageJ* processing software (NIH; v.1.45). For each immunoreactive axon varicosity, an unlabeled profile was randomly selected on the same photomicrograph and the long and short axes, as well as cross-sectional area were measured. Varicosities and myelinated axons were then categorized as containing or not a mitochondrion. For varicosities that showed a synaptic junctional complex, the length of the synaptic junction was measured, the synapse categorized as symmetrical or asymmetrical and the target identified.

The synaptic incidence observed from single-thin sections represents the proportion of examined axon varicosity profiles that exhibit a synaptic contact. The formula of Beaudet and Sotelo (1981) allows the prediction of seeing a synapse if there is one on every varicosity. It takes into account the average size of varicosity profiles, using the long axis as diameter (Umbriaco et al., 1994), the length of their junctional complexes and the thickness of the section. The synaptic incidence extrapolated to the whole volume of varicosities was inferred by comparison to this predicted value, a procedure that was experimentally validated by Umbriaco et al. (1994). Calculation of the g-ratio provided a measure of the degree of axon myelinization. It was calculated by dividing the short axis of the axon without taking into account the myelin sheath by the short axis that includes myelin. g-Ratio obtained for TH-immunostained axons were compared to unlabeled myelinated axons randomly selected from the surrounding neuropil.

### 2.7. Statistics

The statistical Wilcoxon-signed rank test was used to determine differences in the density of TH-immunoreactive axon varicosities between pallidal sectors in the anteroposterior, dorsoventral and lateromedial axes, as well as between entire GPe and GPi. The same statistical approach was used to assess differences in neuronal densities between anteroposterior, dorsoventral and lateromedial sectors and between the GPe and GPi. Statistical differences in dimensions and synaptic incidence between TH-immunoreactive and unlabeled axon varicosities and between TH-immunoreactive axon varicosities in the GPe and GPi were identified by One-Way ANOVA followed by Tuckey's multiple comparison tests. Statistical significance was set at *P* < 0.05 and all analyses were done using GraphPad Prism software (v. 6.0; GraphPad Software, San Diego, CA, USA). Mean and standard error of the mean are used throughout the text as central tendancy and dispersion measures.

## 3. Results

### 3.1. Two types of TH-immunoreactive fibers in the monkey GPe and GPi

**Figure 1:**
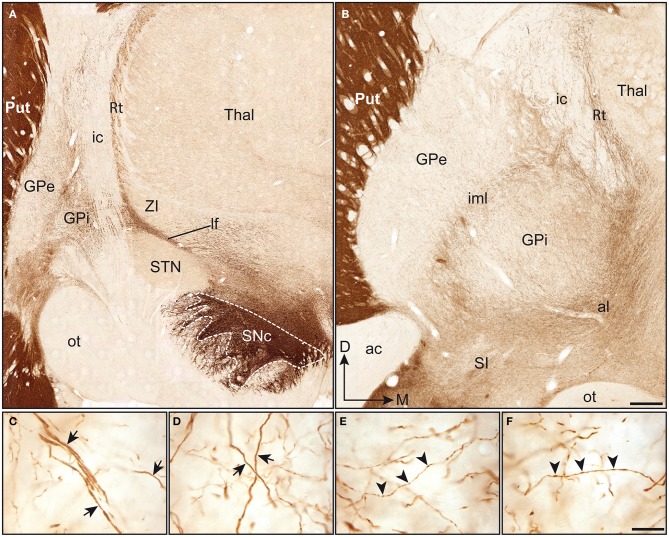
**Coronal sections immunstained for TH and taken through the pallidum at a posterior (A) and anterior (B) level**. Note that the TH immunoreactivity is particularly high in the putamen (Put), when compared to the pallidal complex (GPe-GPi). In **(A)**, immunoreactive axons emerge mediodorsally to the substantia nigra pars compacta (SNc). At this posterior level, TH+ axons run within the lenticular fasciculus (lf), between the subthalamic nucleus (STN) and the zona incerta (ZI), and pierce the internal capsule (ic) to invade the pallidal complex. At a more anterior level **(B)**, TH+ fibers are seen to enter the pallidal complex by coursing within the ansa lenticularis (al) and to invade the pallidum from its ventral surface. At this particular level, the intensity of TH immunostaining appears slightly higher in the GPi than in the GPe. Thick and smooth TH+ axons (arrows) are observed in both the GPe **(C)** and the GPi **(D)** and follow a dorsoventral or lateromedial course across pallidal segments. Thinner axons bearing small and fusiform TH+ axon varicosities (arrowheads) are also seen in abondance in the GPe **(E)** and the GPi **(F)**. Scale bars: 1 mm **(A,B)** and 20 μm **(C–F)**.

The TH positive (+) axons innervating the pallidal complex in squirrel monkeys derive from a massive fiber bundle that emerges mediodorsally to the SNc and courses rostrally within the lateral hypothalamic area. At a posterior level, TH+ axons run within the lenticular fasciculus, along the dorsal surface of the subthalamic nucleus, pierce the internal capsule, where they display a typical band-like pattern, and invade the pallidum from its dorsal surface (Figure 1A). At an anterior level, TH+ fibers sweep laterally along the ansa lenticularis and invade the pallidum from its ventral surface (Figure 1B). These labeled fibers intertwine within the accessory, internal and external medullary laminae, and arborize profusely in the GPe and GPi.

Although much less densely innervated than the adjoining striatum, the pallidum does nevertheless harbor a significant number of heterogeneously distributed TH+ axons and axon varicosities. When comparing the two pallidal segments at the anterior level, the TH immunoreactivity appears less intense in the GPe than in the GPi (Figure 1B). However, a careful examination of the pallidum along its entire anteroposterior extent indicates more subtle regional variations in the density of TH+ elements, as detailed below. Examination at higher magnification reveals the presence of two types of TH+ axons within the squirrel monkey pallidum. Axons of the first type are characteristically thick and smooth, possibly corresponding to myelinated axons ultimately destined to the striatum. These fibers meander throughout the two pallidal segments by loosely following ventrodorsal or mediolateral routes (Figures 1C,D). Many of them reach the putamen by coursing within the external medullary lamina. The thick and smooth fibers, which abound particularly in the ventral portion of the GPe and GPi, reach the caudate nucleus by piercing the internal capsule. Axons of the second type are thinner than those of the first type and they typically display small fusiform axon varicosities (Figures 1E,F). Their trajectories within the pallidum are essentially similar to those of the larger fibers, and both types of axons are often closely intermingled with one another. Overall, TH+ fibers appear less numerous in the GPe than in the GPi and the proportion of the two types of labeled axons varies between pallidal segments, as well as from one region to another. While axons of the first and second type occur in equal proportions at anterior levels in the GPe, the GPi displays a majority of thick and smooth axons. By contrast, at more posterior levels, a majority of TH+ axons in the GPe are thick and smooth, while the axons of the first and second type occur in about equal proportions in the GPi.

### 3.2. Heterogeneous regional distribution of TH-immunoreactive axon varicosities

**Figure 2:**
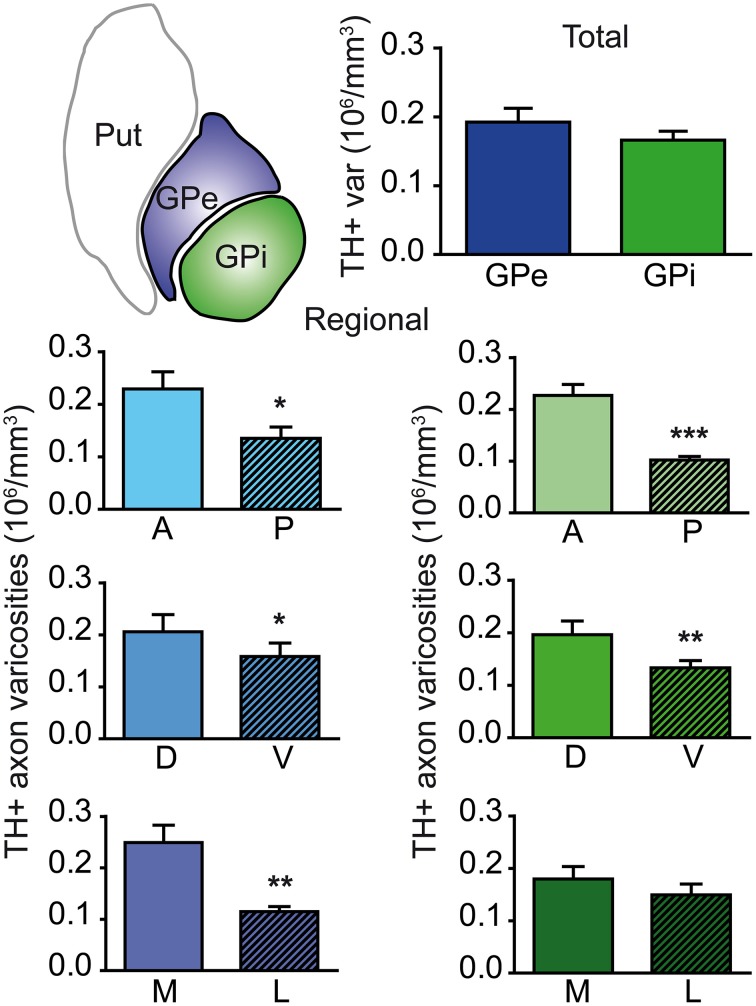
**Histograms showing the density of TH+ axon varicosities in the monkey pallidum**. Average numbers are given for the entire GPe and GPi (upper histogram), as well as for the anterior (A), posterior (P), dorsal (D), ventral (V), medial (M) and lateral (L) sectors of the GPe (left histograms, blue) and for the coresponding sectors of the GPi (right histograms, green). Data for the density of TH+ axon varicosities are expressed in million (10^6^) of axon varicosities per mm^3^ of tissue. ^***^P < 0.001, ^**^P < 0.01 and ^*^P < 0.05, by Wilcoxon signed-rank test.

Axon varicosities displaying TH immunoreactivity were clearly visible on each section (Figures 1C–F), and thus their number could easily be estimated by means of the unbiased quantification method described above. Stereological estimates reveal that the GPe contains twice as many TH+ axon varicosities (3.9 ± 0.3 × 10^6^) than the GPi (1.9 ± 0.1 × 10^6^). However, this figure has to be corrected for the fact that the GPe is nearly two times more voluminous than the GPi (20.2 ± 0.3 vs. 11.4 ± 0.6 mm^3^), as estimated by Cavalieri's method. When this correction is applied, the density of TH innervation, as expressed in millions of axon varicosities per volumetric unit of tissue, is about the same in the two pallidal segments, that is 0.19 ± 0.02 × 10^6^ TH+ axon varicosities/mm^3^ of tissue in the GPe vs. 0.17 ± 0.01 × 10^6^/mm^3^ in the GPi (Figure 2). Although similar in density, TH+ axon varicosities are distributed according to highly heterogeneous topographical patterns in the two pallidal segments. For example, the labeled axon varicosities follow a statistically significant anteroposterior decreasing gradient in both the GPe (*P* = 0.02) and the GPi (*P* = 0.001). The mean density value of TH+ axon varicosities in the rostral half of the pallidum is 0.24 ± 0.01 × 10^6^ for the GPe and 0.23 ± 0.01 × 10^6^ axon varicosities/mm^3^ for the GPi, compared to 0.14 ± 0.02 × 10^6^ and 0.10 ± 0.01 × 10^6^/mm^3^ for the same two structures in their posterior half. A significant decreasing gradient of TH+ innervation also exists along the dorsoventral axis of the GPe (0.22 ± 0.01 × 10^6^ dorsally vs. 0.17 ± 0.01 × 10^6^ axon varicosities/mm^3^ ventrally, *P* = 0.03) and the GPi (0.21 ± 0.01 × 10^6^ dorsally vs. 0.14 ± 0.01 × 10^6^ axon varicosities/mm^3^ ventrally, *P* = 0.01). Moreover, the density of TH+ innervation also decreases along the mediolateral axis, but this variation reaches statistical significance only in the GPe (*P* = 0.002), with a mean value of 0.29 ± 0.02 × 10^6^ in the medial half of the GPe compared to 0.12 ± 0.01 × 10^6^ axon varicosities/mm^3^ in its lateral counterpart (Figure 2). Hence, the antero-dorso-medial portion in the two pallidal segments is markedly richer in TH+ axon varicosities than the other pallidal sectors.

### 3.3. Innervation of GPe and GPi neurons by TH-immunoreactive axons

**Figure 3:**
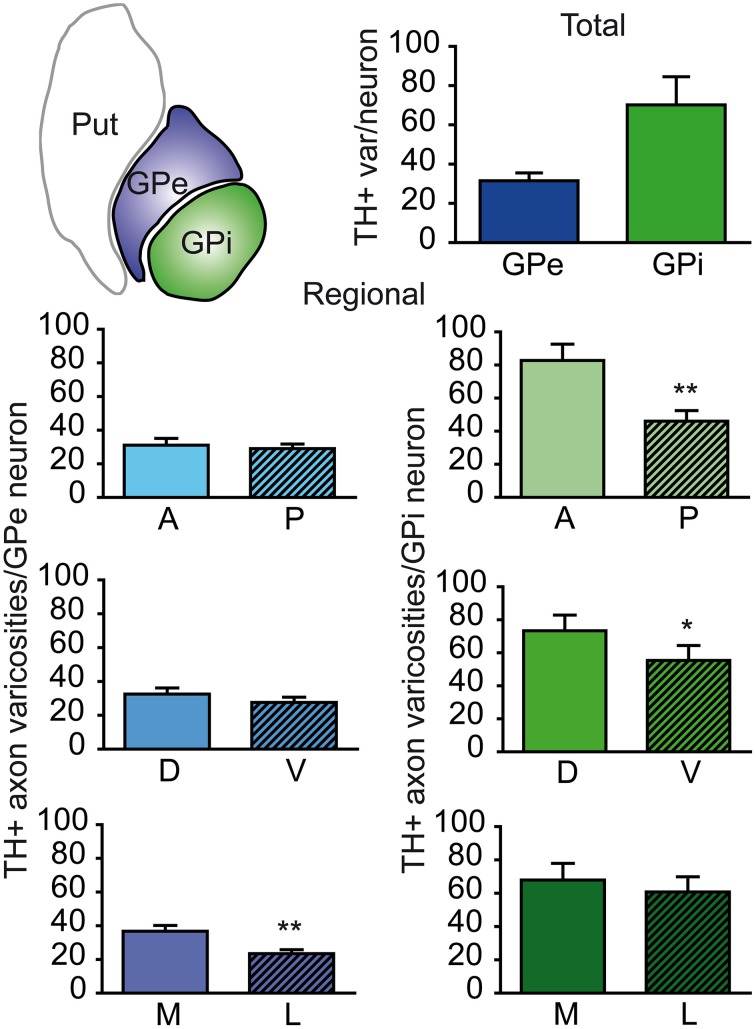
**Histograms showing the number of TH+ axon varicosities per pallidal neuron**. Average numbers are given for the entire GPe and GPi (upper histogram), as well as for the anterior (A), posterior (P), dorsal (D), ventral (V), medial (M) and lateral (L) sectors of the GPe (left histograms, blue) and for corresponding sectors of the GPi (right histograms, green). ^**^P < 0.01 and ^*^P < 0.05, by Wilcoxon signed-rank test.

The neuronal population of the GPe and GPi was assessed stereologically on Nissl-stained sections. The GPe harbors 80,287 ± 10,160 neurons, compared to 26,265 ± 5325 neurons in the GPi. When corrected for the fact that the GPe is about twice as large as the GPi, the neuronal density values are rather similar in the two pallidal segments: 3.47 ± 0.15 × 10^3^ neurons/mm^3^ of tissue in the GPe compared to 2.70 ± 0.18 × 10^3^ in the GPi. When these values are combined with our estimates of the number of TH+ axon varicosities, it becomes possible to determine the number of TH+ axon varicosities per pallidal neuron and thus to assess the relative strength of the DA innervation on a single typical pallidal neuron. Based on our statistical analysis, there appear to be no significant difference between the GPe and GPi in regard to the number of TH+ axon varicosities per pallidal neuron, the overall values being 28 ± 3 TH+ axon varicosities/GPe neuron compared to 68 ± 15 TH+ axon varicosities/GPi neuron (Figure 3). However, we noted some regional variations between the GPe and GPi in respect to the density of TH innervation at single neuronal level. For example, the number of TH+ axon varicosities remains relatively constant along the anteroposterior and dorsoventral axes in the GPe, whereas significant decreasing gradients are found along the same planes in the GPi. The anterior and posterior halves of the GPe contain 28 ± 4 and 28 ± 3 TH+ axon varicosities/neuron, respectively, whereas the corresponding values for the GPi are 83 ± 17 and 44 ± 11 (*P* = 0.002). Similarly, the dorsal and ventral halves of the GPe harbor 29 ± 3 and 26 ± 3 TH+ axon varicosities/neuron, respectively, compared to 78 ± 14 and 55 ± 15 TH+ axon varicosities/neuron in the GPi (*P* = 0.02). In contrast, the number of TH+ axon varicosities/neuron varies significantly along the mediolateral axis of the GPe (33 ± 4 vs. 22 ± 2; *P* = 0.004), but remains relatively constant in the GPi (73 ± 16 vs. 61 ± 14; Figure 3).

### 3.4. Fine morphological features and asynaptic character of TH-immunoreactive innervation

**Figure 4:**
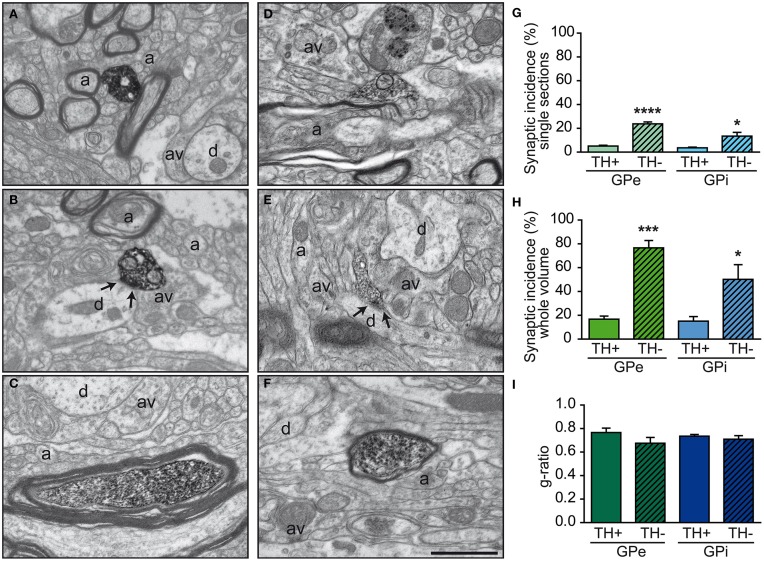
**Examples of TH+ axon varicosities and myelinated axons in the GPe (A–C) and GPi (D–F) as visualized by electron microscopy after labeling with the immunoperoxidase-diaminobenzidine technique**. TH+ axon varicosities observed in both GPe **(A,B)** and GPi **(D,E)** are usually surrounded by small axons (a), either myelinated or not, and unlabeled axon varicosities (av). Small dendritic profiles (d) are also often found in the surrounding microenvironment, as seen in **(A** and **E)**. The TH+ axon varicosities observed in **(B** and **E)** establish a symmetric synaptic contact (between arrows) with a dendritic profile (d). Histograms of synaptic incidence observed in single-thin sections **(G)** and extrapolated to the whole volume of varicosities **(H)** indicate that in both the GPe and GPi, very few of the TH+ axon varicosities are engaged in synaptic contact, compared to their unlabeled counterparts (TH-). TH immunostaining is also found in numerous myelinated axons, as shown in **(C** and **F)**. TH+ myelinated axons have the same degree of myelination as their unlabeled congeners (TH-), as indicated by similar g-ratio **(I)**. ^****^P < 0.0001, ^***^P < 0.001, ^*^P < 0.05 between TH+ and unlabeled axon varicosities, by One-Way ANOVA. Scale bar: 1 μm.

The TH+ axon varicosities observed within the GPe and GPi typically have their axoplasm filled with diaminobenzidine which also lines the plasma membrane and the outer surface of organelles. They are generally ovoid, contain aggregated small and clear vesicles and frequently display one or more mitochondria (Figures 4A,B,E,F). The TH immunostaining within the GPe and GPi is also frequently observed in large myelinated axons filled with mitochondria, probably belonging to axons *en passant* heading for the putamen and the caudate nucleus (see Figures 4C,D).

Comparisons of morphological measurements reveal that TH+ axon varicosities in the GPe are significantly smaller than those in the GPi, and that the latter are larger than the randomly selected unlabeled profiles, as measured by their long axis [*F*_(3, 12)_ = 17.90, *P* < 0.0001], diameter [*F*_(3, 12)_ = 13.06, *P* = 0.0004], cross-sectional area [*F*_(3, 12)_ = 7.872, *P* = 0.004], and aspect ratio [*F*_(3, 12)_ = 5.286, *P* = 0.01; Table 1). Indeed, GPe TH+ axon varicosities display a significantly smaller long axis (0.78 ± 0.03 μm) and diameter (0.63 ± 0.03 μm) than the GPi TH+ axon varicosities (0.99 ± 0.02 μm, *P* = 0.0003 and 0.75 ± 0.01 μm, *P* = 0.001, respectively, by Tuckey's *post-hoc* test). The smaller size of GPe TH+ axon varicosities is also assessed by a smaller cross-sectional area (0.35 ± 0.04 μm^2^) than for GPi TH+ axon varicosities (0.48 ± 0.02 μm^2^, *P* = 0.007, by Tuckey's *post-hoc* test). Moreover, the TH+ axon varicosities observed in the GPe are rounder (1.67 ± 0.02, aspect ratio) than the TH+ axon varicosities found in the GPi (1.97 ± 0.05, *P* = 0.02, by Tuckey's *post-hoc* test). Along with these morphological differences between GPe and GPi TH+ axon varicosities, variations in morphometrical features also exist between TH+ axon varicosities and their unlabeled counterparts, but these differences reach statistical significance only in the GPi where the TH+ axon varicosities are larger than their unlabeled counterparts (Table 1). The average long axis of TH+ axon varicosities in the GPi is 0.99 ± 0.02 μm compared to 0.83 ± 0.03 μm for their unlabeled counterparts (*P* = 0.003, by Tuckey's *post-hoc* test) and the average diameter size is 0.75 ± 0.01 μm for TH+ axon varicosity profiles compared to 0.66 ± 0.01 μm for unlabeled profiles (*P* = 0.01, by Tuckey's *post-hoc* test). The larger size of the TH+ axon varicosities in the GPi is also attested by a significantly larger cross-sectional area (0.48 ± 0.02 μm^2^) than for the unlabeled varicosity profiles (0.37 ± 0.02 μm^2^; *P* = 0.03, by Tuckey's *post-hoc* test).

**Table 1:** **Morphometric features of TH-immunostained vs. randomly selected unlabeled axon varicosity profiles in the monkey external and internal pallidum**.

	**GPe (*n = 4*)**		**GPi (*n = 4*)**	
	**TH**	**Unlabeled**	**TH**	**Unlabeled**
Number examined	195	183	220	200
Dimension				
Short axis (μm)	0.47±0.02	0.47±0.01	0.50±0.01	0.48±0.00
Long axis (μm)	0.78±0.03^†††^	0.76±0.02	0.99±0.02^**^	0.83±0.03
Aspect ratio	1.67±0.02^†^	1.68±0.08	1.97±0.05	1.83±0.08
Diameter (μm)	0.63±0.03^††^	0.62±0.01	0.75±0.01^**^	0.66±0.01
Area (μm^2^)	0.35±0.04^††^	0.34±0.02	0.48±0.02^*^	0.37±0.02
% with mitochondria	63±4	79±5	73±9	85±13

*Data are presented as mean ± SEM. The unlabeled profiles were selected at random from the same micrograph displaying TH profiles, as explained in Section 2*.

††† *P < 0.001*,

†† *P < 0.01*,

† P < 0.05 for GPe vs. GPi and

** *P < 0.01*,

* *P < 0.05 for TH vs. unlabeled*.

In both GPe and GPi, TH+ axon varicosities observed in single sections are rarely seen to establish genuine synaptic contacts, especially when compared to their unlabeled counterparts [F_(3, 12)_ = 25.71, *P* < 0.0001]. As seen in ultrathin sections, only 5 ± 1% of TH+ axon varicosities in the GPe display an area of synaptic membrane specialization compared to 24 ± 2% for their unlabeled counterparts (*P* < 0.0001, by Tuckey's *post-hoc* test). Likewise, the proportion of synaptic contacts in the GPi is 4 ± 1% for TH+ axon varicosities compared to 13 ± 3% for their unlabeled counterparts (*P* = 0.01, by Tuckey's *post-hoc* test; Figure 4G). Using the parameters validated by Umbriaco et al. (1994) along with the stereological formula of Beaudet and Sotelo (1981), we extrapolated the synaptic incidence to the whole volume of varicosities and estimated that a significantly smaller proportion of TH+ axon varicosities in the GPe and GPi are endowed with a synaptic junction compared to unlabeled axon varicosities [F_(3, 12)_ = 16.32, *P* = 0.0002]. We estimate that only 17 ± 3% of GPe TH+ axon varicosities display a synaptic contact compared to 77 ± 6% for their unlabeled counterparts (*P* = 0.0004, by Tuckey's *post-hoc* test). This also holds true for the GPi, where the synaptic incidence is 15 ± 4% for TH+ axon varicosities compared to 50 ± 12% for their unlabeled counterparts (*P* = 0.02; see Figure 4H). The few synaptic contacts established by TH+ axon varicosities in the GPe and GPi target exclusively pallidal dendrites, indicating that modulation of pre-synaptic elements occurs mainly through volume transmission of DA. These scarce synaptic contacts are of the symmetrical and asymmetrical type in equal proportions in the GPi, whereas more symmetrical synapses are found in the GPe (Table 2). In both pallidal segments, TH+ myelinated axons harbor a significantly larger proportion of mitochondria (109 ± 5 and 95 ± 3% in the GPe and GPi, respectively) compared to their unlabeled counterparts (53 ± 9 and 48 ± 7%, respectively; *P* < 0.0001). Their degree of axon myelination was calculated as the g-ratio, whose value increases when the thickness of the myelin decreases. Such a calculation reveals that the TH+ myelinated axons coursing through both pallidal segments display a similar degree of myelination (0.77 ± 0.04 in the GPe and 0.74 ± 0.01 in the GPi) to that of their unlabeled counterparts (0.68 ± 0.05 and 0.71 ± 0.03, respectively). In addition, the degree of myelination of the TH+ axons is similar in the GPe and GPi (0.77 ± 0.04 vs. 0.74 ± 0.01; Figure 4I).

**Table 2:** **Junctional characteristics of TH-immunostained vs. randomly selected unlabeled axon varicosity profiles in the monkey external and internal pallidum**.

	**GPe (*n = 4*)**		**GPi (*n = 4*)**	
	**TH**	**Unlabeled**	**TH**	**Unlabeled**
Synaptic incidence (%)				
Single section	5±1^****^	24±2^††^	4±1^*^	13±3
Whole volume	17±3^***^	77±6	15±4^*^	50±12
Length of synaptic
junction (μm)	0.25±0.00	0.25±0.01	0.27±0.06	0.23±0.03
Junctions (%)				
Symmetrical	75±16	96±3	46±21	90±7
Asymmetrical	25±16	3±3	54±21	10±7

*Data are from the same sectional varicosity profiles as in Table 1 (mean ± SEM). The varicosity profiles were classified as showing or not a synaptic junction according to the criteria described in Section 2. The synaptic incidence for the whole volume of varicosities was extrapolated from the formula of Beaudet and Sotelo (1981), using the long axis as diameter of profiles (Umbriaco et al., 1994)*.

**** *P < 0.0001*,

*** P < 0.001 and

* P < 0.05 for TH vs. unlabeled and

†† *P < 0.01 for GPe vs. GPi*.

## 4. Discussion

The morphological, topographical, and ultrastructural data gathered in the present study has shed a new light on the anatomical substratum whereby DA exerts its influence on the primate pallidum. Our light microscopic investigation has revealed that DA axon terminals are distributed throughout the entire extent of the GPe and GPi according to a heterogeneous pattern that characterizes each of the two pallidal segments. These results were complemented by a detailed ultrastructural analysis showing that DA acts upon both the GPe and GPi neurons and might use a volumic mode of transmission to exert its influence on pallidal neurons and their major afferents. These findings provide new insights on the involvement of the ascending DA projection in the functional organization of the primate GPe and GPi. These two morphologically similar nuclei occupy markedly different positions in the motor-related subcortical microcircuitry, the GPe being a key integrator and the GPi a major output structure of the basal ganglia. The functional significance of the morphological data gathered in the present study will now be discussed in the light of the current literature.

### 4.1. Density and morphological features of DA axons in the GPe and GPi

The primate pallidum was previously shown to display a much lower density of DA innervation than the adjoining putamen and caudate nucleus (Lavoie et al., 1989; Sutoo et al., 1994; Porritt et al., 2005), but was reportedly more densely innervated by DA axons than the subthalamic nucleus (Lavoie et al., 1989), in agreement with the data gathered here in squirrel monkeys. However, there are some inconsistencies in the results of previous studies where the density of the DA innervation of the two pallidal segments was compared in human and non-human primates. Some investigations in the squirrel monkey reported the DA axons to be less densely arborized in the GPe than in the GPi (Parent and Smith, 1987; Lavoie et al., 1989), whereas both pallidal segments were found to be similarly innervated by DA axons in the vervet monkey (*Cercopithecus aethiops*) and the human (Jan et al., 2000). In another human postmortem investigation, the GPe was described as being more densely innervated than the GPi (Porritt et al., 2005). These inconsistencies may reflect some interspecific variations, but they are most likely the result of differences in the various methodological approaches that were used in these studies, which were essentially qualitative in nature. A further confounding factor is the presence of two types of TH+ fibers at pallidal levels: thick and smooth axons, which are more likely fibers of passage en route to the striatum, and thin axons displaying vesicle-filled varicosities, which are the elements that interact specifically with pallidal neurons and their afferents, as shown in the present study. These two types of TH+ axons, whose presence have been noted in both rodents and primates (Rodrigo et al., 1998; Jan et al., 2000; Prensa et al., 2000; Fuchs and Hauber, 2004; Debeir et al., 2005), have not been clearly distinguished from one another in the pioneering studies of the pallidal DA innervation cited above. In the present investigation, we focussed essentially on the TH+ axon varicosities present at pallidal levels and, with the help of unbiased stereological procedures, we were able to provide the first detailed quantitative analysis of the DA innervation of the primate pallidum. Our data clearly reveal that, although the GPi appears to contain more immunoreactive axons than the GPe, the density of DA axon varicosities is fairly similar between the two pallidal segments of the squirrel monkeys. This can be explained by the fact that the GPi displays a larger number of thick and smooth fibers and because the axons found in the GPe are more varicose than those observed in the GPi. Likewise, there is no statistically significant difference between the GPe and the GPi in regard to the number of TH+ axon varicosities per pallidal neuron.

### 4.2. Topographical arrangement of the DA axon varicosities within the GPe and GPi

The present study has provided the first stereologically-based evidence for the fact that the density of DA innervation is similar in the two segments of the primate pallidum, as determined by the number of TH+ axon varicosities per volumetric unit of pallidal tissue or per single pallidal neuron. Yet, despite such a similarity, significant variations were noted between the two pallidal segments in respect to the regional distribution of the DA axon varicosities. For example, our estimates of the density of TH+ axon varicosities per mm^3^ of pallidal tissue reveal significant anteroposterior and dorsoventral decreasing gradients in both pallidal segments, whereas mediolateral decreasing gradient occurs only in the GPe. However, when the density of the DA pallidal innervation is evaluated in terms of the number of TH+ axon varicosities per single pallidal neuron, such anteroposterior and dorsoventral decreasing gradients remain significant only in the GPi, while the number of TH+ axon varicosities/neuron decreases along the mediolateral axis in the GPe, but remains constant in the GPi.

Previous studies have shown the DA innervation of the GPe and GPi in squirrel monkeys (Lavoie et al., 1989) and humans (Jan et al., 2000) to be distributed according to an anteroposterior decreasing gradient, but such regional variations were not reported in vervet monkeys (Jan et al., 2000) and rats (Fuchs and Hauber, 2004). At variance with the present findings, however, a previous study in squirrel monkeys (Lavoie et al., 1989) reported a mediolateral decreasing gradient in the DA innervation of the GPi, while the present investigation in the same species reveals that such a gradient exists, but only in the GPe. As mentioned above, this type of discrepancy might simply reflect the fact that the evaluation of the density of the DA innervation in these earlier studies was largely based on the qualitative assessment of heterogeneous neuronal elements (e.g., thick and smooth vs. thin and varicose fibers), whereas the present account is essentially the result of stereological estimates of the number of DA axon varicosities.

The functional significance of such topographical heterogeneities is difficult to ascertain, but some insights might be gained by examining the pattern of pallidal DA innervation in the context of three major functional territories of the primate pallidum. The associative, sensorimotor, and limbic cortical areas are known to project in a segregated manner onto three distinct regions of the striatum, referred as the associative, sensorimotor, and limbic striatal territories (see review by Parent, 1990; Parent and Hazrati, 1995). As a result of the topographical organization of the striatofugal projections, these three functional modalities are largely maintained at pallidal levels. For example, striatal neurons located in the associative territory project to most of the GPe at anterior commissure levels and to the dorsomedial third of the GPe and GPi caudal to the anterior commissure, whereas neurons in the sensorimotor territory target mainly the ventrolateral two-thirds of the post-commissural GPe and GPi. Neurons located in the limbic striatal territory project principally to the so-called ventral pallidum and the medial tip of the GPi (Smith and Parent, 1986; Parent, 1990; Saint-Cyr et al., 1990; Hedreen and DeLong, 1991; Hazrati and Parent, 1992; Flaherty and Graybiel, 1994; François et al., 1994). In the present study, the pallidal DA innervation was found to be more dense in the anterior and dorsal sectors of both GPe and GPi, as well as in the medial half of the GPe. These DA-rich regions appear to correspond principally to the associative and, to a lesser extent, the sensorimotor pallidal territories. These findings suggest that DA acts principally upon pallidal neurons that are under the influence of associative and sensorimotor striatal neurons, which are involved, respectively, in the preparation and execution of motor responses (Parent, 1990; Hedreen and DeLong, 1991; Flaherty and Graybiel, 1994).

However, such interpretation must be tempered by the fact that DA modulation of pallidal neurons does not depend only on the number of DA axon varicosities, but is markedly influenced by the density and location of DA receptors, at both regional and cellular levels. For instance, when examined topographically, DA receptors of the D_1_ and D_5_ types appeared rather homogeneously distributed in the primate GPi, but a closer analysis at the single neuronal level reveals that the vast majority of D_1_ receptors occur on unmyelinated axons, whereas most D_5_ receptors are confined to pallidal cell bodies and proximal dendrites (Kliem et al., 2010). Such specific receptor distribution might allow DA to act directly through synaptic transmission upon GPi neurons via the D_5_ receptor and indirectly through volumic transmission by modulating the release of GABA by striatopallidal fibers through the D_1_ receptor. A limiting factor regarding the functional territoriality of the primate pallidum is the remarkable length of the massively innervated pallidal dendrites, which can reach up to 1 mm (Fox et al., 1974; DiFiglia et al., 1982; Yelnik et al., 1984). Being mostly oriented along the dorsoventral axis, these long pallidal dendrites very often extend over two distinct pallidal territories, which renders them capable of integrating neuronal information originating from more than one functional territory of the striatum (Flaherty and Graybiel, 1994; François et al., 1994; Parent and Hazrati, 1995). Single-cell anatomical and electrophysiological studies of the primate GPe and GPi would significantly further our understanding of the way DA influences the parallel or funelling type of neural processing that occurs at pallidal levels.

### 4.3. Ultrastructural features of DA axon varicosities in the pallidum

The present ultrastructural investigation has revealed that the DA axon varicosities present in the squirrel monkey GPe and GPi are larger than those previously described in other areas of the primate brain, such as the dorsal and ventral striatum (Smith et al., 1994; Ikemoto et al., 1996), the thalamus (Melchitzky et al., 2006; García-Cabezas et al., 2009) and the prefrontal cortex (Martin and Spühler, 2013). Furthermore, the DA axon varicosities in the squirrel monkey GPe were found to be smaller than those in the GPi. The overall larger size of pallidal DA axon varicosities compared to those in the striatum is congruent with the hypothesis of a distinct nigropallidal pathway in primates (Smith et al., 1989; Parent et al., 1990; Jan et al., 2000), whereas the fact that DA axon varicosities in the GPe are smaller than those in the GPi raises the possibility of a distinct DA innervation of each pallidal segments (Parent and Smith, 1987; Parent et al., 1990; Charara and Parent, 1994; Jan et al., 2000). However, possible methodological variations between the present and earlier ultrastructural studies, as well as the putative influence of postsynaptic targets on the determination of the morphological features of presynaptic elements must be taken into account so as to validate these conclusions.

The present study has provided the first detailed quantitative analysis of the ultrastructural features of the pallidal DA innervation in primates. It has allowed us to document, among other things, the existence of genuine DA synaptic contacts, which occur essentially upon pallidal dendrites and are of both the symmetrical and asymmetrical types. This finding reveals that, in addition to the indirect effect it exerts upon pallidal neurons by modulating the activity of striatofugal neurons, DA is able to act directly upon GPe and GPi neurons through synaptic interactions mediated by the D_2_-like and D_1_-like families of DA receptors, respectively, (see Kliem et al., 2010). Well-characterized DA synaptic contacts of the symmetrical and asymmetrical types were also detected in the primate thalamus and nucleus accumbens (Ikemoto et al., 1996; García-Cabezas et al., 2009), whereas only symmetrical DA synapses were observed in the rat striatum (Descarries et al., 1996). The presence of DA synapses of both symmetrical and asymmetrical types in the primate pallidum, together with a heterogeneous mixture of excitatory D_1_-like and inhibitory D_2_-like receptors, suggest that DA is able to exert both excitatory and inhibitory effects upon pallidal neurons and their afferents. These features also explain the dual effect often observed following pallidal infusion of DA receptor agonists and antagonists (Qi and Chen, 2011).

Our study reveals that the vast majority of the DA axon varicosities observed in the primate pallidum are devoid of synaptic specialization: only 15–20% of the TH+ axon varicosities in the GPe and GPi were engaged in synaptic relationship with pallidal neurons by comparison with 50–75% of their unlabeled congeners. Yet, although the majority of pallidal DA axon varicosities are asynaptic, they all harbor a multitude of synaptic vesicles, a morphological trait that underlies their capacity to release transmitter (Marchbanks, 1979; Volknandt, 1995). Such a morphological organization may favor a presynaptic DA modulation of the primate pallidum, as it appears to be the case in the globus pallidus (Cooper and Stanford, 2001; Querejeta et al., 2001) and ventral pallidum (Mengual and Pickel, 2002) of rodents. In primates, the major DA effect on pallidal neurons is likely the result of presynaptic events occurring upon striatopallidal axons, which account for more than 90% of all pallidal afferents (Parent and Hazrati, 1995) and are enriched in DA receptors of the D_1_ and the D_2_ types (Gerfen and Bolam, 2010). Such a view is supported by the results of numerous electro-pharmacological studies undertaken in both rodents and primates (see Rommelfanger and Wichmann, 2010). In monkeys, for example, the activation of receptors of the D_2_ type with specific agonists induces: (a) an increase in the firing rate of GPe neurons, most likely due to a blockade of striatopallidal inhibitory inputs, and (b) a decrease in the activity of GPi neurons, possibly resulting from a D_2_-mediated effect on glutamatergic afferents, which are immunoreactive for the D_2_-like receptors (Hadipour-Niktarash et al., 2012). Likewise, the activation of receptors of the D_1_ type with specific agonists leads to a decrease in discharge rates of GPi neurons accompanied by an increase in the local release of GABA, whereas opposite effects are observed with D_1_ antagonists (Kliem et al., 2007, 2010). Despite a thorough electron microscopic examination of the entire GPe and GPi of the squirrel monkey, we were unable to detect axo-axonic contacts involving a TH-labeled element. Such a finding indicates that the DA presynaptic modulation of the various pallidal afferents described above is likely to be exerted in a paracrine manner, that is, through a signaling molecule that acts at a certain distance from its relase site. Such mode of action of DA, which is often referred to as *volumic transmission*, is far from being unusual, as it is reportedly occurring in various other brain areas of rodents and primates, such as the prefrontal cortex (Martin and Spühler, 2013), the striatum (Arluison et al., 1984; Descarries et al., 1996; Bérubé-Carrière et al., 2012), the nucleus accumbens (Ikemoto et al., 1996), and the thalamus (Melchitzky et al., 2006; García-Cabezas et al., 2009).

### 4.4. Functional considerations

Despite its relatively modest size compared to the robust striatopallidal and subthalamopallidal projections, the functionality of the DA pallidal projection and its impact on the basal ganglia has been documented by numerous electrophysiological, pharmacological and behavioral studies undertaken under both normal and pathological conditions (see Fuchs and Hauber, 2004; Björklund and Dunnett, 2007; Rommelfanger and Wichmann, 2010). In rodents, DA was shown to play a primary role in modulating the firing rates and patterns of pallidal neurons involved in motor control (Ruskin et al., 2001; Karain et al., 2015). For example, injections of D_1_ and D_2_ receptor agonists in the rodent GP produced akinesia (Hauber and Lutz, 1999), whereas DA infusion into the GP partially restored motor deficits in a rat model of Parkinson's disease (Galvan et al., 2001). In primates, glial-cell-line-derived neurotrophic factor (GDNF) was shown to induce sprouting of DA axons in the GPe and SNc of monkeys rendered Parkinsonian following 1-methyl 4-phenyl 1,2,3,6-tetrahydro pyridine (MPTP) intoxication, a phenomenon that was correlated with a functional recovery of motor symptoms (Gash et al., 1996). Furthermore, electrophysiological studies revealed that the loss of pallidal DA innervation participates in the development of the typical bursting mode discharge and changes in firing rates that occur in the GPe and GPi of Parkinsonian monkeys (Filion and Tremblay, 1991; Filion et al., 1991; Boraud et al., 1998).

In accordance with the heterogeneous feature of the nigrostriatal DA pathway revealed by single-axon labeling (Gauthier et al., 1999; Prensa and Parent, 2001), immunohistochemical observations in Parkinsonian monkeys have suggested that this projection is composed of several subsystems, each having a specific cellular origin, a distinct axonal terminal territory and a different degree of vulnerability to MPTP (Parent et al., 1990; Parent and Lavoie, 1993). The two extremes of such a morphological continuum are: (a) the DA subsystem that arises in the ventral tier of the SNc and terminates in the sensorimotor striatal territory, wich appears highly sensititive to MPTP, and (b) the DA projection that emerges from the VTA and aborizes in the ventral striatum, which is resistant to the neurotoxin (Parent and Lavoie, 1993). The DA projection that arises principally from the dorsal tier of the SNc and terminates within the pallidum was found to occupy a somewhat intermediary position in what appears to be relatively spared in Parkinsonian monkeys (Parent et al., 1990; Schneider and Dacko, 1991; Parent and Lavoie, 1993; Mounayar et al., 2007; Dopeso-Reyes et al., 2014). Similar findings obtained in Parkinsonian patients were taken as an indication that the preserved DA nigropallidal projection might be involved in some compensatory mechanims (Whone et al., 2003). However, other data gathered in both Parkinsonian patients and monkeys have revealed significant alterations in the DA innervation of the pallidum (see review by Benazzouz et al., 2014), whereas other investigations have suggested that the preservation of the DA nigropallidal projection occurs only in the early phases of the disease (Whone et al., 2003; Mounayar et al., 2007).

Obvioulsy more studies are needed to better understand the role of the DA innervation of pallidal neurons in the functional organization of the basal ganglia in both normal and pathological conditions. Up to now, the data we have gathered in the squirrel monkey suggest that, by virtue of their predominantly volumic mode of action, the DA, serotoninergic and cholinergic brainstem ascending systems (Eid et al., 2013, 2014) exert a collaborative modulatory influence upon pallidal neurons in concert with the more direct GABAergic inhibitory and glutamatergic excitatory actions of the striatum and subthalamic nucleus. They further reveal that, in addition to the action they exert at striatal levels upon the cell bodies at the origin of the striatopallidal projections, nigral DA neurons have a direct access to pallidal neurons of the GPe, which is a key integrative component of the basal ganglia, as well as to neurons of the GPi, which is a major output structure of the basal ganglia.

## Author contributions

LE contributed to the conception and design of the experiments, conducted all the experiments, acquisition, analyses and data interpretation and wrote the manuscript. MP is the principal investigator who designed the study and revised the manuscript. Both LE and MP approved the final version of the manuscript and agreed to be accountable for all aspects of the work.

### Conflict of interest statement

The authors declare that the research was conducted in the absence of any commercial or financial relationships that could be construed as a potential conflict of interest.

## Abbreviations

a: axon
ABC: avidin–biotine complex
ac: anterior commissure
al: ansa lenticularis
AP: anteroposterior
av: axon varicosity
d: dendrite
DA, dopamine: dopaminergic
DAT: dopamine transporter
GABA: gamma-aminobutyric acid
GDNF: glia-cell-line-derived neurotrophic factor
GPe: external segment of the globus pallidus
GPi: internal segment of the globus pallidus
ic: internal capsule
iml: internal medullary lamina
lf: lenticular fasciculus
MPTP, 1-methyl 4-phenyl 1, 2, 3: 6-tetrahydro pyridine
ON: overnight
ot: optical tract
PB: phosphate-buffered saline
PBS: sodium phosphate-buffered saline
Put: putamen
Rt: reticular thalamic nucleus
SI: substantia innominata
SNc: substantia nigra pars compacta
STN: subthalamic nucleus
TBS: tris-buffered saline
TH: tyrosine hydroxylase
Thal: thalamus
ZI: zona incerta.
