# Corrigendum: Morphological evidence for dopamine interactions with pallidal neurons in primates

**DOI:** 10.3389/fnana.2015.00127

**Published:** 2015-09-29

**Authors:** Lara Eid, Martin Parent

**Affiliations:** Department of Psychiatry and Neuroscience, Centre de Recherche de l'Institut Universitaire en Santé Mentale de Québec, Université LavalQuebec City, QC, Canada

**Keywords:** basal ganglia, globus pallidus, squirrel monkey, electron microscopy, stereology, pallidum, substantia nigra, TH immunohistochemistry

In the abstract, one should read ≪ Estimation of the neuronal population in the GPe (3.47 ± 0.15 × 10^3^ neurons/mm^3^) and GPi (2.69 ± 0.18 × 10^3^) ≫ instead of ≪ Estimation of the neuronal population in the GPe (3.47 ± 0.15 × 10^3^ neurons/mm^3^) and GPi (2.69±0.18 × 10^6^) ≫.

Original article has been updated.

## Conflict of interest statement

The authors declare that the research was conducted in the absence of any commercial or financial relationships that could be construed as a potential conflict of interest.

