# Supplementary material for: Morphological evidence for dopamine interactions with pallidal neurons in primates
Source: Front Neuroanat. 2015 Aug 11;9:111. doi: 10.3389/fnana.2015.00111 (PMC4531254; doi:10.3389/fnana.2015.00111)
Supplement: Supplementary file 1 [file Image1.PDF]

Supplementary Material: Morphological evidence for dopamine interactions with pallidal neurons in primates

Lara Eid and Martin Parent *

*Correspondence:
Author Name: Martin Parent, PhD
martin.parent@crulrg.ulaval.ca

1 SUPPLEMENTARY TABLES AND FIGURES

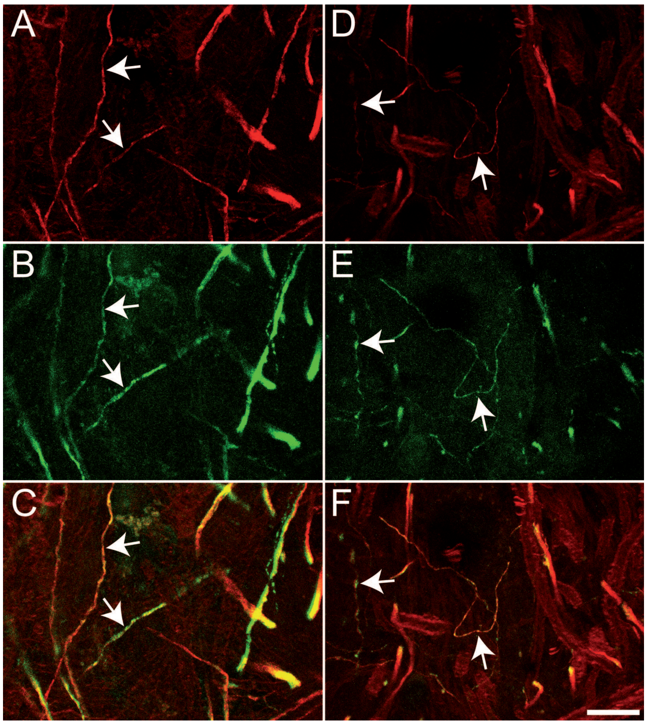

Figure 1. High magnification views of TH+ (**A**, **D**) and DAT+ (**B**, **E**) axons, as seen with the confocal microscope, in the GPe (**A**-**C**) and GPi (**D**-**F**) of squirrel monkeys. Note that all TH+ fibers are immunoreactive for DAT (**C**, **F**, see arrows for examples), indicating that TH+ axons and axon varicosities analyzed in the present study are dopaminergic. Scale bar : 20 μm.
